# Supplementary material for: Stochastic resonance of rotating particles in turbulence
Source: Nat Commun. 2025 Nov 24;16:10376. doi: 10.1038/s41467-025-65316-8 (PMC12644856; doi:10.1038/s41467-025-65316-8)
Supplement: Supplementary file 1 — Supplementary information [file 41467_2025_65316_MOESM1_ESM.pdf]

Supplementary Information: Stochastic resonance of rotating particles in turbulence

Ziqi Wang,[1] Xander M. de Wit,[1] Roberto Benzi,[2,3,✉] Chunlai Wu,[1]
Rudie P. J. Kunnen,[1] Herman J. H. Clercx,[1] and Federico Toschi[1,4,✉]

[1] *Fluids and Flows group and J.M. Burgers Center for Fluid Mechanics, Department of Applied Physics and Science Education, Eindhoven University of Technology, 5600 MB Eindhoven, Netherlands*

[2] *Sino-Europe Complex Science Center, School of Mathematics North University of China, Shanxi, Taiyuan 030051, China*

[3] *Department of Physics and Istituto Nazionale di Fisica Nucleare, University of Rome Tor Vergata, Rome I-00133, Italy*

[4] *Consiglio Nazionale delle Ricerche - Istituto per le Applicazioni del Calcolo, Rome I-00185, Italy*

(Dated: October 8, 2025)

I. EXPERIMENTAL SETUP

A. Particle Characterization

In optical images as shown in Fig. 1, the particle surfaces may appear "rough." This appearance originates from the contrast between the bright Styrofoam core and the darker magnetic coating, rather than actual geometric protrusions. Variations in paint deposition, the side of the particle in contact with the substrate during spraying, and small inconsistencies in the magnetic paint spraying angle result in non-uniform coating patterns. These patterns enhance optical contrast, creating the visual impression of surface roughness. However, the actual geometric irregularities are negligible compared to the particle diameter, and the surfaces remain effectively smooth on the hydrodynamic scale. Therefore, while such patterns may introduce minor perturbations to local flow, they do not significantly alter translational drag or rotational resistance beyond the effects already captured by particle size and magnetic torque.

The particles are designed to probe rotational dynamics under the combined influence of turbulence and a rotating magnetic field. Their size is comparable to the Kolmogorov scale, ensuring interactions with the smallest turbulent structures while remaining much smaller than the integral scale and Taylor microscale. Consequently, they cannot be regarded as point-like; however, finite-size effects on the rotation are limited, and the dominant particle–flow interactions occur at dissipative scales. The particles' magnetic properties were characterized using a Superconducting Quantum Interference Device (SQUID) magnetometer (Quantum Design Inc.). Batch measurements yielded a mean volume susceptibility of $|\overline{\chi}_{\mathrm{V}}| = 0.136 \pm 0.046$, while single-particle measurements revealed an average anisotropy of $\Delta\chi = 0.011 \pm 0.002$.

B. Magnetic field calibration

The rotating planar magnetic field was calibrated using a SENIS F3A magnetic-field-to-voltage transducer with an integrated 3D Hall probe. Across the frequency range of 1–50 Hz, the maximum magnetic flux density remained stable at 1.60 ± 0.02 mT. The driving frequency is set to be 20 Hz for Fig. 2 and 10 Hz for Fig. 4, which illustrates the stochastic resonance. Further details of the field characterization are reported in our experimental study [1].

C. Flow field characterization

Our experiments are conducted in a Von Kármán-type turbulent flow, characterized by a Taylor-scale Reynolds number of up to $\mathrm{Re}_\lambda = 447$, ensuring fully developed homogeneous and isotropic turbulence in the measurement volume. It is noteworthy that the particle rotational dynamics is dominated by the turbulent dynamics at the small scales, i.e. the scales of vortex filaments. It is widely conjectured, motivated by numerical and experimental observations, that the small scales of turbulence are universal [2–6]. Therefore, it is expected that, provided the

✉ roberto.benzi@gmail.com
✉ f.toschi@tue.nl

turbulence is fully developed, the strength of the large scale forcing, i.e., different values of the Reynolds number, poses negligible effects on the particle rotational dynamics. While the background flow is turbulent, the particle rotational dynamics are primarily governed by interactions with small-scale eddies. The particle rotational Reynolds number, $\mathrm{Re_p} = \rho_\mathrm{f} r^2 |\omega_\mathrm{p} - \omega_\eta|$, is estimated to be $\mathcal{O}(10^{-1})$ (below 1) based on typical maximum relative angular velocities, justifying the use of the laminar (Stokes) rotational drag coefficient, ξ_r in our theoretical modelling. This ensures that viscous forces dominate inertial effects at the particle scale, providing an accurate leading-order description of particle-fluid interaction.

While the Von Kármán-type turbulent flow is not perfectly homogeneous and isotropic, a fully developed turbulent flow can be generated in the central region of the container with small-scale statistics close to local isotropy [7–13]. This region, measuring approximately 20 mm × 20 mm × 20 mm, serves as the primary domain for measurements. Since our small particles ($r \sim 5\eta$) primarily interact with dissipative-scale velocity gradients in this central region, the observed stochastic resonance and symmetry breaking are governed by particle-scale turbulence and magnetic forcing rather than large-scale flow anisotropy.

The measured dissipation rate ε is determined from the driving torque on the two impellers as $\varepsilon = T\Omega/(\rho V_\mathrm{w})$ [14, 15], where T is the sum of the driving torques from the impellers at the top and bottom of the water tank and $V_\mathrm{w} = 2.8 \times 10^{-3}$ m^3 is the volume of the water tank. The driving torques are measured using two strain gauge torque meters mounted on the motors. In our experiments, we investigated two turbulence intensities: (1) the weak-turbulence condition, for which the results are shown in Fig. 2(b–d) of the main text; and (2) the strong-turbulence condition, for which the results are presented in Supplementary Fig. 1(b). For the weak-turbulence case, the impellers rotate at 0.83 Hz. The corresponding mean energy dissipation rate is $\varepsilon = 0.037 \pm 0.002$ m^2/s^3, which yields a Taylor-scale Reynolds number of $\mathrm{Re}_\lambda = 398$, a Kolmogorov length scale of $\eta = 0.072$ mm, and a Kolmogorov time scale of $\tau_\eta = 5.20$ ms. The corresponding particle Stokes number is $\mathrm{St} = d_\mathrm{p}^2/(12\beta_\mathrm{p}\nu\tau_\eta) = 4.36$. For the strong-turbulence case, the impellers rotate at 1.21 Hz. The corresponding mean energy dissipation rate is $\varepsilon = 0.075 \pm 0.001$ m^2/s^3, which yields a Taylor-scale Reynolds number of $\mathrm{Re}_\lambda = 447$, a Kolmogorov length scale of $\eta = 0.061$ mm, and a Kolmogorov time scale of $\tau_\eta = 3.66$ ms. The corresponding particle Stokes number is $\mathrm{St} = 6.08$.

II. COMPARISON BETWEEN EXPERIMENTAL AND NUMERICAL RESULTS

Previous studies have indeed explored the rotational dynamics of magnetically driven objects in quiescent environments [16–24], demonstrating phenomena such as phase locking and oscillatory motion. However, a fundamental distinction of our work lies in investigating these dynamics within the stochastic and chaotic environment of fully developed turbulence. This critical inclusion reveals a new class of phenomena, particularly the emergence of stochastic resonance where turbulent fluctuations play an active role in enhancing the particle response to an external periodic magnetic field. Furthermore, we uncover a novel symmetry-breaking mechanism for inducing net particle rotation in zero-mean turbulent vorticity.

In the main paper, we have presented selected comparisons of the PDFs of particle angular velocity, $\omega_{\mathrm{p},z}$, between experimental and numerical results (Fig. 2 in the main paper), for weak (1.2 mT), **c** intermediate (1.4 mT), and **d** strong (1.6 mT) magnetic intensities with constant rotational frequency of the magnetic field (20 Hz), to illustrate the key findings. The PDFs are obtained using 3000 (in experiments) and 512 (in simulations) particle trajectories. Here, we provide additional comparisons to further support our conclusions.

Supplementary Fig. 1 presents the probability density distributions of the particle angular velocity components $\omega_{\mathrm{p},i}$ ($i = x, y, z$) obtained from both experiments and numerical simulations. In Supplementary Fig. 1a (Supplementary Movie 9), where no magnetic field is applied, the particle rotation is purely driven by turbulence, exhibiting isotropic behavior. To facilitate a direct comparison with the experimental results (circles), the numerical results (squares) are flattened over all directions (i.e., reshape the angular velocity vector into a one-dimensional (1D) array while preserving the original data order). In Supplementary Fig. 1b (Supplementary Movie 10), when a magnetic field of 1.6 mT (same intensity as that of Fig. 2d in the main paper) is applied and the turbulence intensity is increased ($\mathrm{Re}_\lambda = 447$), the numerical and experimental results show good agreement, demonstrating that the numerical simulation is capable of capturing the essential physics of the experiment. Supplementary Fig. 1c further provides a phase diagram illustrating different particle rotational dynamic regimes, which is reproduced from Fig. 3m in the main paper. The experimental conditions corresponding to Supplementary Fig. 1b are marked as a green triangle. As shown in Supplementary Figures 1a and b, the simulation results agree well with the experimental results. The slight discrepancies are due to the polydispersity of the particles. Specifically, these particles exhibit anisotropy in three key aspects: (1) Shape: The particles are nearly spherical, with an average diameter of $d_\mathrm{p} = 0.762 \pm 0.066$ mm. However, minor deviations from perfect sphericity exist due to natural variations in the manufacturing process. These variations can introduce small differences in rotational dynamics, particularly when particles experience complex hydrodynamic and magnetic interactions in turbulence. (2) Density: The mean density of the magnetic particles is measured to be

$\rho_\mathrm{p} = 0.208 \pm 0.014$ kg/m^3. This value is significantly lower than that of water, indicating that the particles remain buoyant in the experimental fluid. The density variation across different particles arises from heterogeneities in the composition of the Styrofoam core and the magnetic coating. (3) Magnetic Properties: The magnetic characteristics of the particles are inherently non-uniform due to the way the magnetic paint adheres to the surface during fabrication. Unlike ideal dipoles, these particles exhibit variability in three crucial aspects: the magnitude of the magnetic moment $|\mathbf{m}|$, the orientation of the magnetic moment relative to the particle's geometric axes, and the spatial distribution of magnetization across the surface. This non-uniformity means that different particles respond to an externally applied magnetic field in distinct ways, leading to variations in their rotational dynamics. Furthermore, the magnetization patterns on the particle surface serve as markers for tracking their rotational motion in experiments. All these three aspects of particle polydispersity can introduce minor differences between the simulation results w.r.t. the experimental observations. However, these differences remain small, and overall, the simulations show good agreement with the experiments.

In all experiments, the particle volume fraction is maintained at 0.17%, ensuring that the system remains in the dilute regime. This low concentration minimizes inter-particle interactions, allowing us to focus on the response of individual particles to turbulent flow and external magnetic fields without significant collision effects.

These additional comparisons reinforce the validity of our numerical model in capturing the stochastic rotational dynamics of the anisotropic magnetic particles under turbulence and external magnetic fields.

III. THEORETICAL MODEL

A. Statistics of direction angle

When analyzing the mechanism of stochastic resonance, we employ a simplified model of the particle rotational dynamics. In this model, the governing equation is reformulated in terms of the phase lag angle β, under the assumption that the angle (α) between the orientation direction ($\mathbf{n}$) and z-axis ($\hat{\mathbf{n}}$) satisfies $\alpha \to \pi/2$, which corresponds to $\kappa = \sin^2\alpha \to 1$. Based on this assumption, the equation simplifies to

$$\dot{\beta} = \omega_H - \frac{\omega_\mathrm{a}}{2}\sin 2\beta - \omega_{\mathrm{f},z}. \tag{S.1}$$

This simplification is justified by the statistical behavior of $\kappa = \sin^2\alpha$, whose probability density function is sharply peaked at 1 and rapidly decays for values smaller than 1. To validate this assumption, we plot the probability density distributions of $\sin^2\alpha$, denoted as $P(\sin^2\alpha)$, for different noise intensities, $\omega_\eta/\omega_\mathrm{a}$, in both the subcritical ($\omega_H/\omega_\mathrm{a} = 0.1 < 1/2$) and supercritical ($\omega_H/\omega_\mathrm{a} = 1.1 > 1/2$) regimes. The results confirm that κ is indeed strongly concentrated around 1 and the probability decreases rapidly for smaller values of α ($< \pi/2$), supporting the validity of our simplification.

B. Overdamped approximation justification

To justify the validity of the overdamped model, we carefully assessed inertial effects. For a spherical particle, the rotational response time can be estimated as $\tau_\mathrm{rot} = \frac{I}{\xi_\mathrm{r}}$, where $I = \frac{2}{5}\mathcal{V}_\mathrm{p}\rho_\mathrm{p}r^2$ is the particle moment of inertia, and $\xi_\mathrm{r} = 8\pi\mu r^3$ is the rotational drag coefficient. Substituting these into the equation for τ_rot simplifies to $\tau_\mathrm{rot} = \frac{\rho_\mathrm{p}r^2}{15\mu}$. Using our experimental parameters of $r_\mathrm{p} = 0.381$ mm, $\rho_\mathrm{p} = 208$ kg/m^3, $\mu \approx 1.0 \times 10^{-3}$ Pa$\cdot$s, and $\tau_\eta = 7.56$ ms. The rotational response time is $\tau_\mathrm{rot} = 2.01$ ms. We observe that τ_rot is approximately $2.01/7.56 = 0.266$ or about one-quarter of τ_η, indicating that the particle rotational dynamics are significantly faster than the characteristic changes in the flow vorticity. Therefore, the particle can respond quickly to the flow vorticity, making the assumption of an overdamped regime a reasonable first approximation. Note that our experimental frequencies are chosen to ensure the particle can follow the driving field within the overdamped regime, i.e., the frequency of the magnetic field ω_H is slower than $1/\tau_{rot} \approx 2000$ Hz, so that the particle rotational motion will remain in the overdamped regime.

C. Neglecting rotation-translation coupling

In the model of particle angular dynamics, we neglect rotation–translation coupling by assuming that the particle center of mass and hydrodynamic center are approximately aligned. This assumption is justified by the nearly spherical

shape, uniform mass distribution, and small size of the particles (compared to the Kolmogorov scale), which collectively minimize translation-induced hydrodynamic torque. Consequently, the hydrodynamic torque induced by translational motion is expected to be negligible. Even if weak coupling exists, its effect can be interpreted as an additional source of rotational noise that is uncorrelated with the external magnetic field. This may slightly modify the amplitude of angular fluctuations or slightly shift the resonance frequency, but it does not alter the underlying mechanism of stochastic resonance. Since the emergence of stochastic resonance in our system fundamentally arises from the balance between external magnetic forcing and intrinsic rotational noise, the presence of small translation–rotation coupling does not qualitatively affect our main conclusions.

D. Validity of the point-particle approximation

Despite neglecting finite-size hydrodynamic effects, the point-particle approximation offers a leading-order description of particle–fluid interaction and is widely adopted in studies of particle-laden flows [25, 26]. In our system, the particle radius is comparable to the Kolmogorov length scale, ensuring locally linear flow gradients that primarily govern rotational dynamics via local vorticity. This, coupled with a dominant external magnetic torque and a dilute suspension (volume fraction of $\Phi_V = 0.17\%$ that minimizes particle-particle interactions [27]), provides a robust physical justification for our model. Crucially, the good agreement between our simulations and experimental measurements further validates the point-particle approximation for this system.

E. Linear stability analysis of the particle rotational dynamics in quiescent fluid

Here we derive theoretically the ideal critical ratio of ω_H/ω_a in a noiseless system and estimate the relaxation time τ^*. We consider the simplified nonlinear equation without the noise term, given by

$$\dot{\beta} = \omega_H - \frac{\omega_a}{2}\sin 2\beta. \tag{S.2}$$

At equilibrium, we set $\dot{\beta} = 0$, which gives $\omega_H - \frac{\omega_a}{2}\sin 2\beta = 0$. If $\left|\frac{2\omega_H}{\omega_a}\right| \leq 1$, the equilibrium points are

$$\beta^* = \frac{1}{2}\arcsin\left(\frac{2\omega_H}{\omega_a}\right) + k\frac{\pi}{2}, \quad k \in \mathbb{Z}. \tag{S.3}$$

Then we introduce a small perturbation around the equilibrium as $\beta = \beta^* + \delta$. Substituting into the governing equation and linearizing the system as $\dot{\delta} = -\omega_a \cos 2\beta^* \cdot \delta$. The solution is $\delta(t) = \delta(0)e^{-\omega_a \cos 2\beta^* t}$.

The stability of the equilibrium is determined by the eigenvalue $\lambda^* = -\omega_a \cos 2\beta^*$. The equilibrium is stable if $\lambda^* < 0$, which requires $\cos 2\beta^* > 0$. Substituting the expression for β^*, we get:

$$\cos\left(\arcsin\frac{2\omega_H}{\omega_a}\right) > 0. \tag{S.4}$$

Using the identity $\cos(\arcsin x) = \sqrt{1-x^2}$, we obtain:

$$\sqrt{1 - \frac{4\omega_H^2}{\omega_a^2}} > 0, \tag{S.5}$$

which implies $|\omega_H| < \frac{\omega_a}{2}$.

The corresponding relaxation time is

$$\tau^* = \frac{1}{|\lambda^*|} = \frac{1}{\omega_a\sqrt{1 - 4\left(\frac{\omega_H}{\omega_a}\right)^2}}. \tag{S.6}$$

Physically, the relaxation time τ^* means the characteristic time it takes for a perturbed rotational motion of the particle to return to its phase-locked state, i.e., $\dot{\beta} = 0$, after a small disturbance of δ.

So if $|\omega_H| < \omega_a/2$, the equilibrium points are stable. If $|\omega_H| > \omega_a/2$, the equilibrium points disappear and the system becomes unstable [20]. Therefore, the critical ratio is $\omega_H/\omega_a = 1/2$. If we use $\omega^* = 1/\tau^*$ as the characteristic relaxation frequency, then we can reformulate Eq. (S.6) as

$$\frac{\omega^*}{\omega_a} = \sqrt{1 - 4\left(\frac{\omega_H}{\omega_a}\right)^2}, \tag{S.7}$$

which is useful when analyzing the mode of the resonance curve later.

IV. WAITING TIME STATISTICS AND EQUIVALENT NOISE INTENSITY ESTIMATION

To gain deeper insight into the stochastic nature of particle rotation in turbulence, we analyze the waiting time, τ, between transitions of the phase lag angle, β, in the absence of an external forcing ($\omega_H = 0$). This allows us to isolate the effect of turbulent vorticity (stochastic fluctuations) on the rotational dynamics of the particle. The waiting time, τ, is defined as the duration (marked by red segments in Supplementary Fig. 3a) between successive transitions of β between its metastable states.

For the light particle case, as shown in Supplementary Fig. 3b, the PDF of τ follows an exponential distribution, $P(\tau) \sim e^{-\lambda\tau}$, where λ represents the characteristic transition rate. This result is consistent with the expected behavior of a noise-driven escape process over an energy barrier, where transitions occur randomly with a Poisson-like distribution. Such Poisson-like distribution behavior is robust for light, neutral, and heavy particles (Supplementary Fig. 3c), with different transition rates, λ, for different types of particles indicating exploring different regions (and thus experiencing different vorticity) in turbulence. The light particles preferentially concentrate in the high vorticity regions [28–30], with stronger turbulence vorticity fluctuations driving β across the energy barrier, leading to a faster transition (higher λ). While the heavy particles tend to concentrate in the low vorticity region and thus a slower transition rate.

The transition rate λ varies with the noise intensity ω_a, as shown in Supplementary Fig. 3d for different types of particles (light, neutral, and heavy). Specifically, λ exhibits an exponential dependence on the normalized potential barrier ω_a/ω_η, namely $\lambda \sim \exp(-\omega_a/\epsilon)$. This behavior is reminiscent of Kramers' escape rate in a double-well potential subjected to thermal noise: given by $\lambda \sim e^{-\Delta U/\epsilon}$ [31–33], where ΔU is the potential barrier and ϵ represents the noise strength. In our case, the effective energy barrier between stable states of β is proportional to ω_a. Physically, ϵ quantifies the relative strength of turbulent fluctuations compared to the deterministic restoring forces acting on the particle. A larger ϵ implies a stronger stochastic influence, facilitating more frequent transitions between metastable states, while a lower ϵ implies that the system remains trapped in its metastable states for longer durations. This aligns with the earlier observation that particles of different densities explore distinct regions of the turbulence and thus different transition rates (slopes) are observed (Supplementary Fig. 3c). The estimated equivalent noise intensity shown in Supplementary Fig. 3e provides a direct means to quantify turbulence-induced noise intensity from observable transition dynamics. Within the investigated parameter space, turbulent fluctuations effectively act as a random forcing term that continuously perturbs the system, leading to memoryless escape events over the effective potential barriers. However, this picture may change if the particle enters the underdamped regime or has a finite size, where inertial effects should be taken into account [26, 34, 35].

Using the equivalent noise intensity ϵ shown in Supplementary Fig. 3e of the light particle (blue symbol), we simulate the behavior of magnetic particles subjected to a rotating magnetic field in the presence of white noise. Specifically, in the governing equation, we replace the turbulent vorticity signal, $\omega_{f,z}$, with a stochastic noise term $\sqrt{\epsilon}\mathcal{N}(0,1)$, with $\mathcal{N}(0,1)$ representing a standard normal distribution with zero mean and unit variance. As shown in Supplementary Fig. 4, the results of the light particles subjected to white noise (Supplementary Fig. 4f-j) are qualitatively similar to those of the light particles in turbulence (Supplementary Fig. 4a-e). This comparison demonstrates that the stochastic transitions observed for the light particles can be effectively captured using a simple white noise model with an appropriately chosen noise intensity ϵ.

V. RESULTS OF HEAVY AND NEUTRAL PARTICLES

In addition to the simulation results presented in the main paper on light particles in turbulence subjected to a rotating magnetic field, we also investigate the dynamics of particles with different densities. Specifically, we analyze neutral particles, characterized by a density contrast parameter $\beta_p = \frac{3}{1+2\rho_p/\rho_f} = 1$ and heavy particles with $\beta_p = 0.01$.

Supplementary Fig. 5 compares the rotational dynamics and stochastic resonance behavior of light, neutral, and heavy particles in a turbulent flow under an applied rotational magnetic field.

The phase diagrams in Supplementary Fig. 5a, f, and k illustrate different rotational regimes: phase-locked, back-and-forth, and turbulent dynamics. A key dynamical transition occurs when $\omega_a = 2\omega_H + \omega_\eta$ for the light particles (Supplementary Fig. 5a) based on the fact that light particles explore the high vorticity region with a fluctuation of the same order as ω_η. While for the heavy particles, because they explore the low vorticity region (smaller than ω_η), the transition boundary is shifted, indicating a wider parameter space for the phase-locked regime in which the particle maintains a stable rotational response to the external field (the purple colored region in Supplementary Fig. 5k). Neutral particles, which distribute more uniformly within the turbulence, exhibit a transition boundary that lies between those of light and heavy particles.

To further explore the stochastic resonance effect, Figures 5b, g, and l present phase diagrams where the color represents the normalized time-averaged derivative of the phase lag, $\langle\dot{\beta}\rangle/\omega_\eta$. A more quantitative analysis of this resonance behavior can be obtained by plotting $\langle\dot{\beta}\rangle/\omega_\eta$ as a function of ω_η/ω_a for different values of ω_H/ω_a. A pronounced resonance peak is observed at $\omega_\eta/\omega_a \approx 1$ for light particles (Supplementary Fig. 5c), ≈ 1.5 for neutral particles (Supplementary Fig. 5h), and ≈ 2 for heavy particles (Supplementary Fig. 5m) when $\omega_H/\omega_a < 1/2$. This shift in resonance frequency reflects the underlying preferential concentration effects [28–30]: lighter particles predominantly experience high-vorticity fluctuations, whereas heavier particles are more likely to sample low-vorticity regions. The resonance peak occurs when the applied magnetic field intensity ω_a is comparable to the turbulence vorticity experienced by the particles. If we define ω_{light}, ω_{neutral}, and ω_{heavy} as the turbulence vorticity experienced by light, neutral, and heavy particles, respectively, and denote $\omega_{\text{a,light}}$, $\omega_{\text{a,neutral}}$, and $\omega_{\text{a,heavy}}$ as the magnetic field intensities at which resonance peaks appear, we find that $\omega_{\text{light}} > \omega_{\text{neutral}} > \omega_{\text{heavy}}$, leading to $\omega_{\text{a,light}} > \omega_{\text{a,neutral}} > \omega_{\text{a,heavy}}$. Given that the resonance curves are normalized by ω_η, the peak positions satisfy

$$\frac{\omega_\eta}{\omega_{\text{a,light}}} < \frac{\omega_\eta}{\omega_{\text{a,neutral}}} < \frac{\omega_\eta}{\omega_{\text{a,heavy}}}. \tag{S.8}$$

For sufficiently large values of ω_η/ω_a, the data collapses onto a universal curve when normalized by ω_H/ω_a, as shown in Supplementary Figures 5d, i, and n. This collapse suggests that the linear response regime, where the turbulence vorticity dominates, is robust across different particle types.

Finally, Supplementary Figures 5e, j, and o show the dependence of the resonance peak location $\left[\frac{\omega_\eta}{\omega_a}\right]_r$ on the ratio ω_H/ω_a. The dashed line represents a fit based on the relaxation frequency, see Eq. (S.7), giving

$$\left[\frac{\omega_\eta}{\omega_a}\right]_r = C\sqrt{1 - 4\left(\frac{\omega_H}{\omega_a}\right)^2}, \tag{S.9}$$

where C is a dimensionless fitting parameter. The excellent agreement between the resonance peak location and this fit underscores the predictive power of the relaxation frequency model.

The resonance peak location $\left[\frac{\omega_\eta}{\omega_a}\right]_r$ is strongly influenced by the proximity to the critical ratio $\omega_H/\omega_a = 1/2$, highlighting the selection of the corresponding relaxation time.

When ω_H/ω_a is significantly smaller than this critical value, the resonance peak location $\left[\frac{\omega_\eta}{\omega_a}\right]_r$ remains almost constant, which is determined by the turbulence vorticity fluctuations experienced by the particles. However, as ω_H/ω_a approaches the critical value, $\left[\frac{\omega_\eta}{\omega_a}\right]_r$ decreases rapidly as the relaxation time diverges. A longer relaxation time allows particles to diffuse out of the potential well even at lower noise levels ω_η/ω_a, explaining the observed shift in resonance peak location.

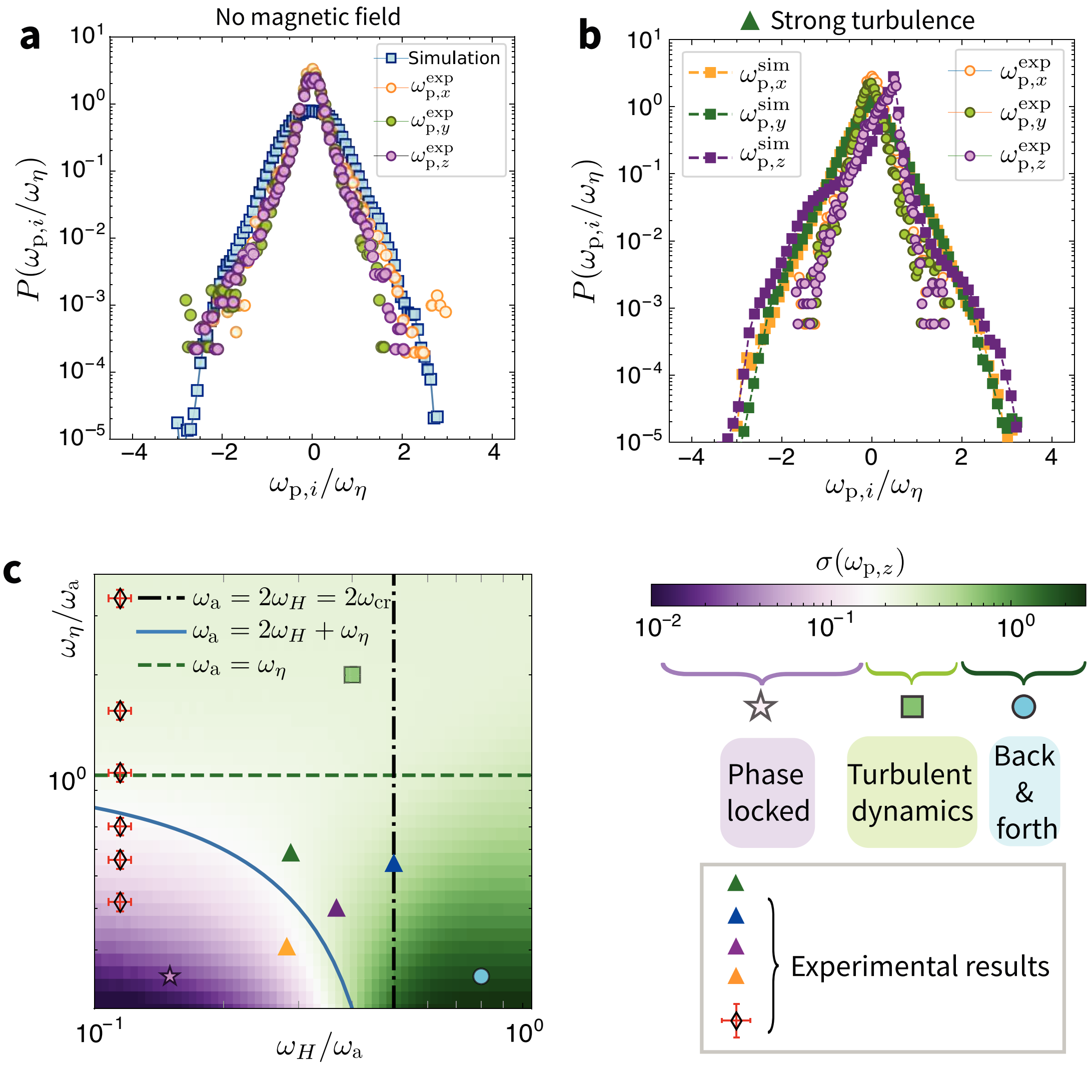

Supplementary Figure 1. **Comparison between experimental and numerical results.** **a** and **b**: The probability density distribution of particle angular velocity $\omega_{p,i}$ with $i = x, y, z$ representing the three components. **a**, In the absence of a magnetic field, the particle rotation is purely driven by turbulence, exhibiting isotropic behavior. The simulation results (squares) are flattened over all directions (i.e., reshape the angular velocity vector into a one-dimensional (1D) array while preserving the original data order) for direct comparison with the experimental results (circles). **b**, With an applied magnetic field (1.6 mT) and increased turbulence (here $Re_\lambda = 447$, while the turbulence used in Fig. 2 of the main paper is $Re_\lambda = 398$), there is a good agreement indicating the numerical simulation can indeed capture the main physics of the experiments when the physical parameters are normalized by the Kolmogrov scales, i.e, ω_η. **c**, Phase diagram of particle rotation dynamic regimes (reproduced from Fig. 3m of the main paper). The experimental setting of **b** is marked as a green triangle.

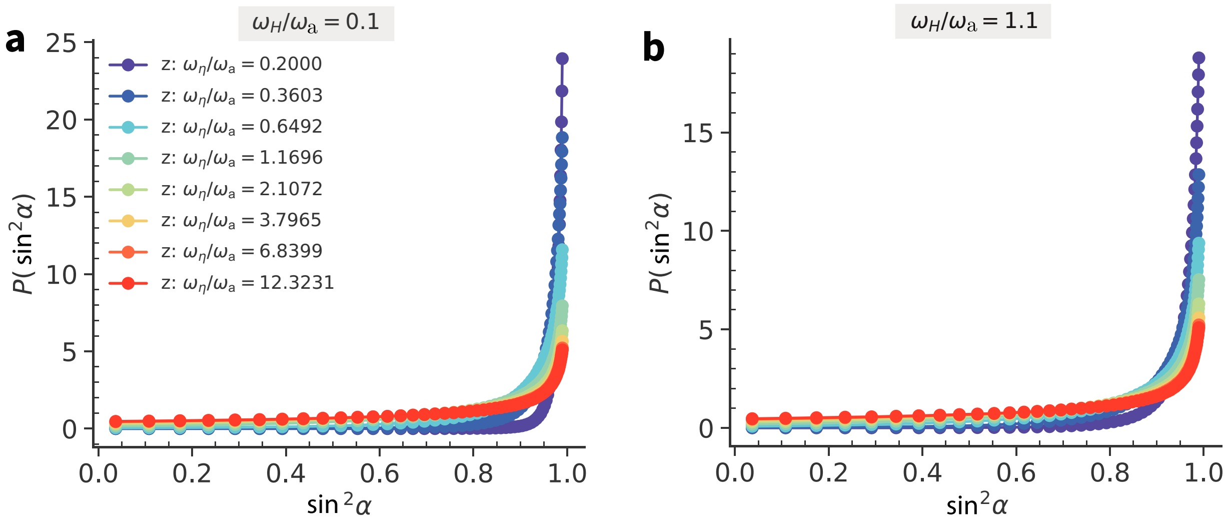

Supplementary Figure 2. **Probability density distribution of** κ**. a**, In the subcritical regime ($\omega_H/\omega_a = 0.1 < 1/2$), the PDFs of $\kappa = \sin^2\alpha$ for different levels of noise intensities (ω_η/ω_a). **b**, In the supercritical regime ($\omega_H/\omega_a = 1.1 > 1/2$), the PDFs of $\sin^2\alpha$ for different levels of noise intensities (ω_η/ω_a). Symbols with the same color correspond to the same value of ω_η/ω_a in **a**.

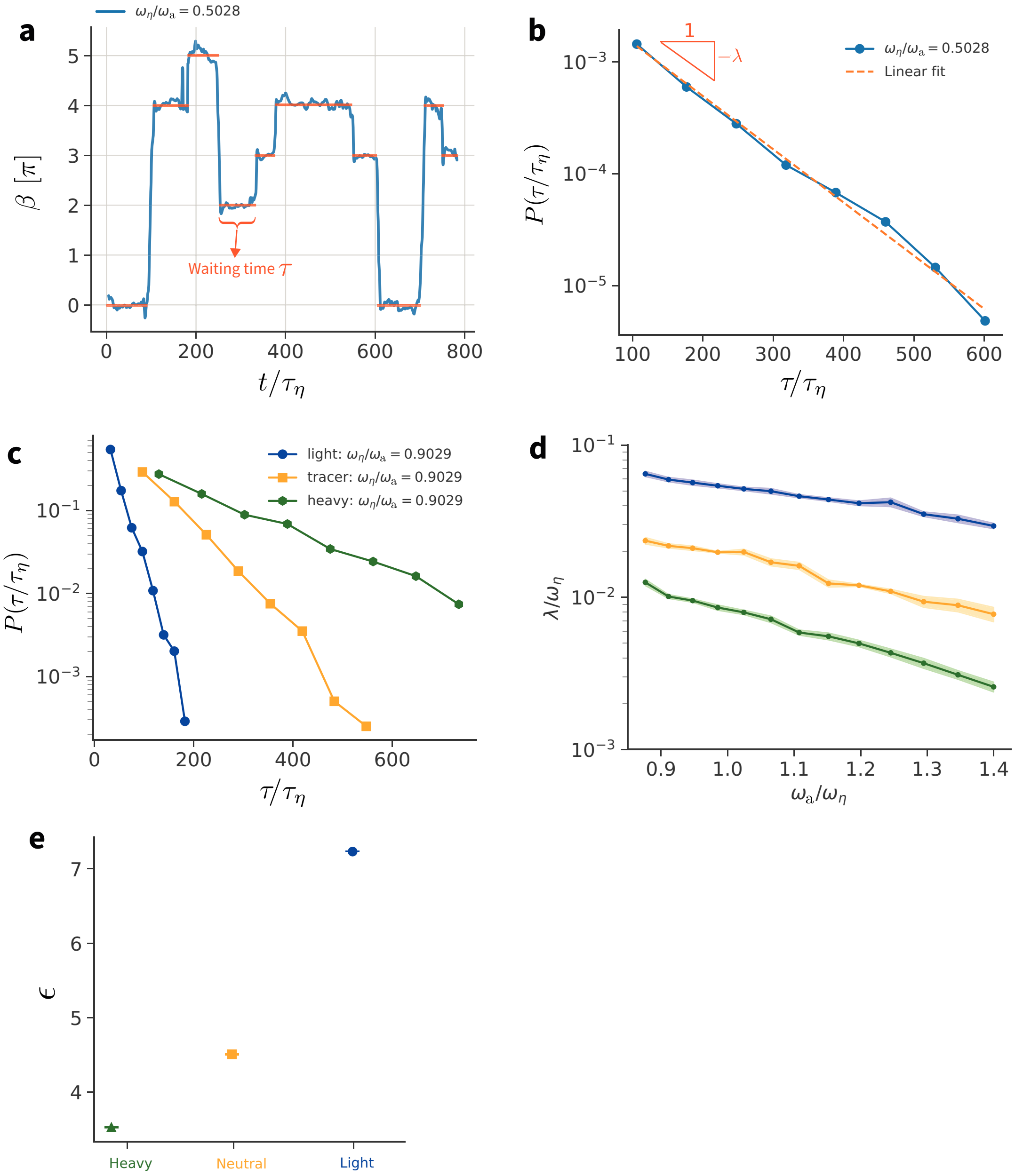

Supplementary Figure 3. **Statistical analysis of waiting time.** The statistical properties of transition time, τ, are analyzed in the absence of external forcing ($\omega_H = 0$), where particle rotational dynamics are solely influenced by turbulent vorticity (stochastic fluctuations). **a**, A typical time series of the phase lag angle, β (blue solid line), with waiting times τ (red segments) marked between transitions. Parameter setting: $\omega_a/\omega_\eta = 2.0$ for a light particle. **b**, The PDF of the normalized waiting time, τ/τ_η. The red dashed line represents an exponential fit, $P(\tau) \sim e^{-\lambda\tau}$ where λ is the characteristic transition rate. **c**, Comparison of the PDF of normalized waiting time, τ/τ_η, for light (blue), neutral (yellow), and heavy (green) particles. The data are collective results of 512 particles for a duration of $12T_L$ (with T_L representing the turbulence integral time scale). The results confirm that $P(\tau) \sim e^{-\lambda\tau}$ holds across different particle types. Parameter setting: $\omega_a/\omega_\eta = 1.1$. **d**, The characteristic transition rate, λ, as a function of the applied noise intensity, ω_a/ω_η, for light (blue), neutral (yellow), and heavy (green) particles. The error bar is indicated by the shaded area, which is the covariance from the fitting process of λ for the relation $P(\tau) \sim e^{-\lambda\tau}$. **e**, The fitted noise intensity, ϵ. Here, ϵ means the equivalent noise intensity in the white noise scenario, i.e., $\omega_{p,z} \sim \sqrt{\epsilon}\mathcal{N}(0,1)$ with $\mathcal{N}(0,1)$ representing the standard normal distribution which has a mean of zero and a variance of one, satisfying the relationship $\lambda \sim e^{-\omega_a/\epsilon}$. The error bar is the covariance from the fitting process of ϵ for the relation $\lambda \sim e^{-\omega_a/\epsilon}$.

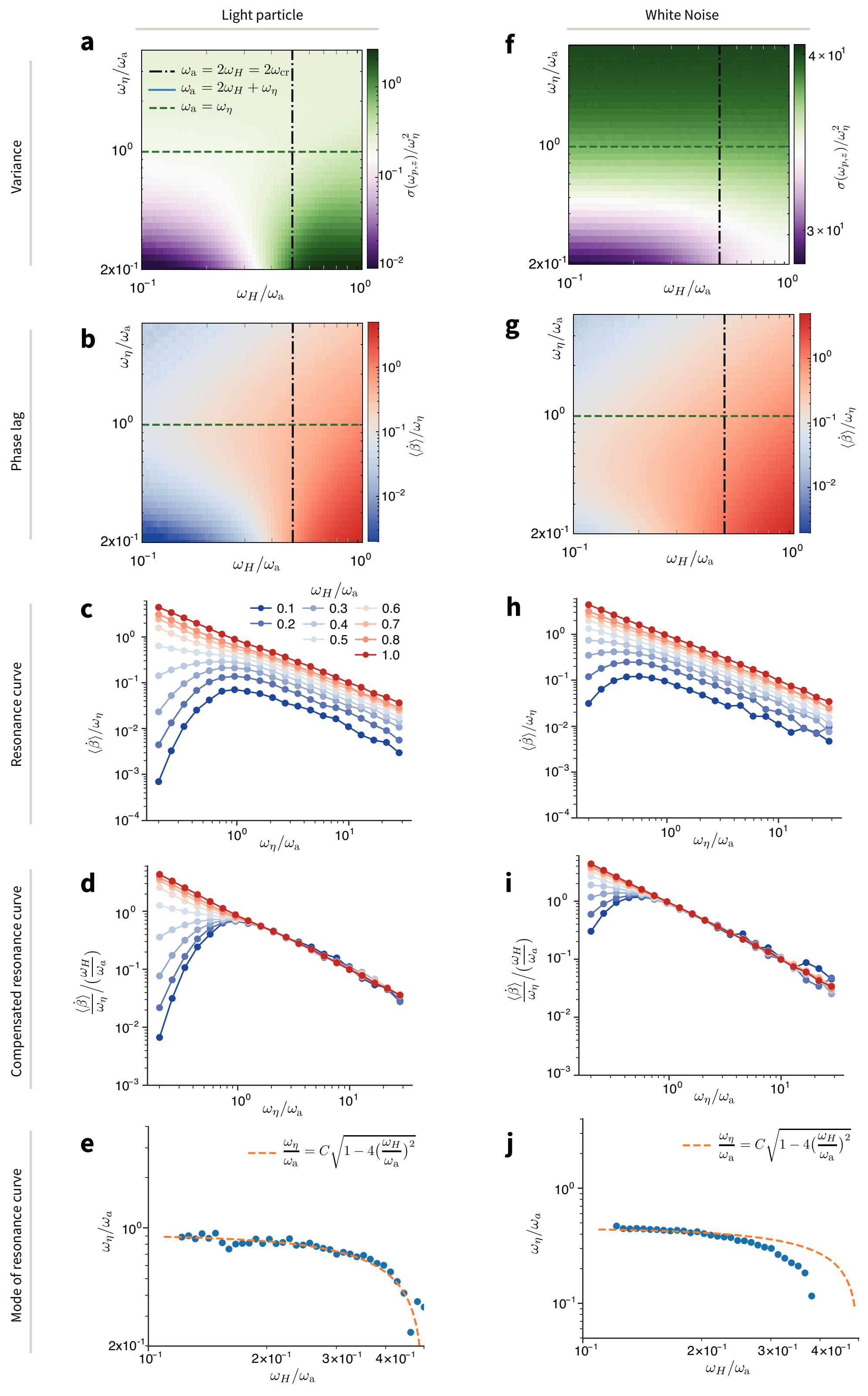

Supplementary Figure 4. **Comparison between the turbulence vorticity signal and white noise.** The white noise term, $\sqrt{\epsilon}\mathcal{N}(0,1)$, in the governing equation of the numerical integration has one key parameter, ϵ, which is the noise intensity. We extract ϵ from the light particle results (namely the value of blue symbol in Supplementary Fig. 3e) and use it as the input parameter for the white noise simulation. The white noise simulation results are shown in **f-j**. For the convenience of comparison, we also show in **a-e** the corresponding results of light particles (namely the results shown in the main paper). **e** and **j**, the mode of the resonance curve shown in **c** and **h** as a function of the ratio ω_H/ω_a. The dashed line is a fitting by Eq. (S.7), i.e., $\omega_\eta/\omega_a = C\sqrt{1-4(\omega_H/\omega_a)^2}$ with C a fitting parameter. C=0.91 (**e**) and 0.45 (**j**).

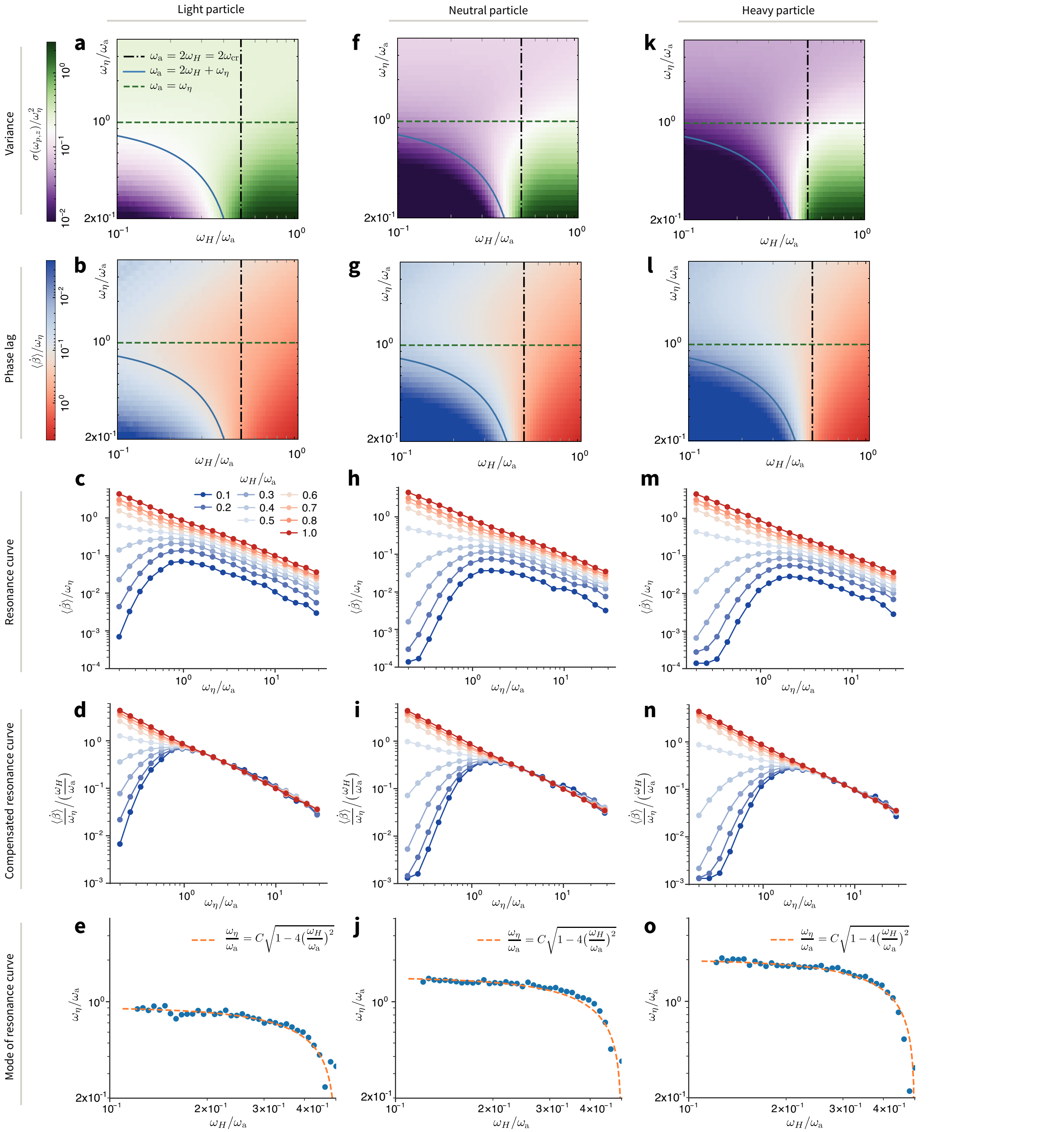

Supplementary Figure 5. Comparison between the light, neutral, and heavy particles. **a**, **f**, and **k**: Phase diagrams of particle rotational dynamics, colored by the normalized variance of the particle angular velocity in the z-direction, $\langle\omega_{p,z}^2 - \langle\omega_{p,z}\rangle^2\rangle/\omega_\eta^2$. The black dot-dashed line indicates the critical frequency, $\omega_{cr} = \omega_a/2$. The green dashed line marks the balance between turbulent fluctuations and magnetic strength, i.e., $\omega_a = \omega_\eta$. The phase-locked regime, predicted by scaling analysis, is enclosed by the thick blue line, satisfying $\omega_a = 2\omega_H + \omega_\eta$. **b**, **g**, and **l**: Phase diagrams of stochastic resonance. The color represents the normalized time-averaged derivative of the phase lag, $\langle\dot{\beta}\rangle/\omega_\eta$. **c**, **h**, and **m**: Plots of $\langle\dot{\beta}\rangle/\omega_\eta$ against ω_η/ω_a for various values of ω_H/ω_a. A pronounced resonant peak emerges at $\omega_\eta/\omega_a \approx 1$ for light particles (**c**), ≈ 1.5 for neutral particles (**h**), and ≈ 2 for heavy particles (**m**) when $\omega_H/\omega_a < 1/2$. A perfect collapse is observed for the linear regime (high ω_η/ω_a) when normalized by ω_H/ω_a (**d**, **i**, and **n**). **e**, **j**, and **o**: The mode of the resonance curve from **d**, **i**, and **n** as a function of the ω_H/ω_a. The dashed line represents a fit base on Eq. (S.7), given by $\omega_\eta/\omega_a = C\sqrt{1-4(\omega_H/\omega_a)^2}$ with fitting parameters: C=0.91 (**e**), 1.5 (**j**), and 2 (**o**).

[1] C. Wu, R. P. J. Kunnen, Z. Wang, X. M. de Wit, F. Toschi, and H. J. H. Clercx, Tracking the rotation of light magnetic particles in turbulence, arXiv preprint arXiv:2506.21769 (2025).
[2] A. Ghira, G. Elsinga, and C. Da Silva, Characteristics of the intense vorticity structures in isotropic turbulence at high reynolds numbers, Physical Review Fluids **7**, 104605 (2022).
[3] S.-J. Kang, M. Tanahashi, and T. Miyauchi, Dynamics of fine scale eddy clusters in turbulent channel flows, Journal of Turbulence **8**, N52 (2007).
[4] M. Tanahashi, S. Iwase, and T. Miyauchi, Appearance and alignment with strain rate of coherent fine scale eddies in turbulent mixing layer, Journal of Turbulence **2**, 006 (2001).
[5] C. B. da Silva, R. J. Dos Reis, and J. C. Pereira, The intense vorticity structures near the turbulent/non-turbulent interface in a jet, Journal of Fluid Mechanics **685**, 165 (2011).
[6] B. Ganapathisubramani, K. Lakshminarasimhan, and N. Clemens, Investigation of three-dimensional structure of fine scales in a turbulent jet by using cinematographic stereoscopic particle image velocimetry, Journal of Fluid Mechanics **598**, 141 (2008).
[7] N. Mordant, E. Lévêque, and J.-F. Pinton, Experimental and numerical study of the lagrangian dynamics of high reynolds turbulence, New Journal of Physics **6**, 116 (2004).
[8] R. Volk, E. Calzavarini, G. Verhille, D. Lohse, N. Mordant, J.-F. Pinton, and F. Toschi, Acceleration of heavy and light particles in turbulence: comparison between experiments and direct numerical simulations, Physica D: Nonlinear Phenomena **237**, 2084 (2008).
[9] G. Zocchi, P. Tabeling, J. Maurer, and H. Willaime, Measurement of the scaling of the dissipation at high reynolds numbers, Physical Review E **50**, 3693 (1994).
[10] G. A. Voth, A. La Porta, A. M. Crawford, J. Alexander, and E. Bodenschatz, Measurement of particle accelerations in fully developed turbulence, Journal of Fluid Mechanics **469**, 121 (2002).
[11] N. Mordant, P. Metz, O. Michel, and J.-F. Pinton, Measurement of lagrangian velocity in fully developed turbulence, Physical Review Letters **87**, 214501 (2001).
[12] A. La Porta, G. A. Voth, A. M. Crawford, J. Alexander, and E. Bodenschatz, Fluid particle accelerations in fully developed turbulence, Nature **409**, 1017 (2001).
[13] R. Volk, E. Calzavarini, E. Leveque, and J.-F. Pinton, Dynamics of inertial particles in a turbulent von kármán flow, Journal of Fluid Mechanics **668**, 223 (2011).
[14] N. Mordant, Characterization of turbulence in a closed flow, J. Phys. II France **7**, 1729 (1997).
[15] R. Labbé, J.-F. Pinton, and S. Fauve, Study of the von kármán flow between coaxial corotating disks, Phys. Fluids **8**, 914 (1996).
[16] V. Croquette and C. Poitou, Cascade of period doubling bifurcations and large stochasticity in the motions of a compass, Journal de Physique Lettres **42**, 537 (1981).
[17] A. Poyé, V. Désangles, X. Jiménez, M. Martin, and Y. Proto, Bipolar motor: rotation, parametric instabilities and chaos, Physica Scripta **94**, 015002 (2018).
[18] A. Kaiser, A. Snezhko, and I. S. Aranson, Flocking ferromagnetic colloids, Science advances **3**, e1601469 (2017).
[19] J. Yan, M. Bloom, S. C. Bae, E. Luijten, and S. Granick, Linking synchronization to self-assembly using magnetic janus colloids, Nature **491**, 578 (2012).
[20] J. Cīmurs, A. Brasovs, and K. Ērglis, Stability analysis of a paramagnetic spheroid in a precessing field, Journal of Magnetism and Magnetic Materials **491**, 165630 (2019).
[21] A. Cēbers and M. Ozols, Dynamics of an active magnetic particle in a rotating magnetic field, Physical Review E **73**, 021505 (2006).
[22] K. Ērglis, Q. Wen, V. Ose, A. Zeltins, A. Sharipo, P. A. Janmey, and A. Cēbers, Dynamics of magnetotactic bacteria in a rotating magnetic field, Biophysical Journal **93**, 1402 (2007).
[23] J. Cīmurs and A. Cēbers, Dynamics of anisotropic superparamagnetic particles in a precessing magnetic field, Physical Review E **87**, 062318 (2013).
[24] K. I. Morozov, Y. Mirzae, O. Kenneth, and A. M. Leshansky, Dynamics of arbitrary shaped propellers driven by a rotating magnetic field, Physical Review Fluids **2**, 044202 (2017).
[25] M. R. Maxey and J. J. Riley, Equation of motion for a small rigid sphere in a nonuniform flow, Physics of Fluids **26**, 883 (1983).
[26] F. Toschi and E. Bodenschatz, Lagrangian properties of particles in turbulence, Annual Review of Fluid Mechanics **41**, 375 (2009).
[27] S. Balachandar and J. K. Eaton, Turbulent dispersed multiphase flow, Annual review of fluid mechanics **42**, 111 (2010).
[28] V. Mathai, D. Lohse, and C. Sun, Bubbly and buoyant particle–laden turbulent flows, Annual Review of Condensed Matter Physics **11**, 529 (2020).
[29] E. Calzavarini, M. Kerscher, D. Lohse, and F. Toschi, Dimensionality and morphology of particle and bubble clusters in turbulent flow, Journal of Fluid Mechanics **607**, 13 (2008).
[30] Z. Wang, X. M. de Wit, and F. Toschi, Localization–delocalization transition for light particles in turbulence, Proceedings of the National Academy of Sciences **121**, e2405459121 (2024).
[31] R. Benzi, Stochastic resonance: from climate to biology, Nonlinear Processes in Geophysics **17**, 431 (2010).
[32] L. Gammaitoni, P. Hänggi, P. Jung, and F. Marchesoni, Stochastic resonance, Reviews of Modern Physics **70**, 223 (1998).

[33] W. Coffey and Y. P. Kalmykov, *The Langevin equation: with applications to stochastic problems in physics, chemistry and electrical engineering*, Vol. 27 (World Scientific, 2012).
[34] R. Zimmermann, Y. Gasteuil, M. Bourgoin, R. Volk, A. Pumir, and J.-F. Pinton, Rotational intermittency and turbulence induced lift experienced by large particles in a turbulent flow, Physical Review Letters **106**, 154501 (2011).
[35] H. Homann and J. Bec, Finite-size effects in the dynamics of neutrally buoyant particles in turbulent flow, Journal of Fluid Mechanics **651**, 81 (2010).
