# Supplementary material for: Stochastic resonance of rotating particles in turbulence
Source: Nat Commun. 2025 Nov 24;16:10376. doi: 10.1038/s41467-025-65316-8 (PMC12644856; doi:10.1038/s41467-025-65316-8)
Supplement: Supplementary file 2 — Description of additional Supplementary Files [file 41467_2025_65316_MOESM2_ESM.pdf]

DESCRIPTION OF ADDITIONAL SUPPLEMENTARY FILES

Supplementary Movie 1: The "phase-locked" regime of a magnetic particle subjected to a rotating magnetic field in a quiescent fluid, corresponding to Fig. 1f in the main paper.

Supplementary Movie 2: The "back-and-forth" regime of a magnetic particle subjected to a rotating magnetic field in a quiescent fluid, corresponding to Fig. 1h in the main paper.

Supplementary Movie 3: The experimental results of the particles immersed in turbulence subjected to a rotating magnetic field with a weak strength, corresponding to Fig. 2b in the main paper.

Supplementary Movie 4: The experimental results of the particles immersed in turbulence subjected to a rotating magnetic field with an intermediate strength, corresponding to Fig. 2c in the main paper.

Supplementary Movie 5: The experimental results of the particles immersed in turbulence subjected to a rotating magnetic field with a strong strength, corresponding to Fig. 2d in the main paper.

Supplementary Movie 6: In the "turbulent-dynamics" regime, the evolution of the tip of the preferred magnetization direction of the particle, $\mathbf{n}(t)$, is visualized in space, corresponding to Fig. 3d in the main paper.

Supplementary Movie 7: In the "phase-locked" regime, the evolution of the tip of the preferred magnetization direction of the particle, $\mathbf{n}(t)$, is visualized in space, corresponding to Fig. 3h in the main paper.

Supplementary Movie 8: In the "back-and-forth" regime, the evolution of the tip of the preferred magnetization direction of the particle, $\mathbf{n}(t)$, is visualized in space, corresponding to Fig. 3l in the main paper.

Supplementary Movie 9: The experimental results of the particles immersed in turbulence without an external magnetic field applied, corresponding to Supplementary Fig. 1a.

Supplementary Movie 10: The experimental results of the particles immersed in a stronger turbulence subjected to a rotating magnetic field, corresponding to Supplementary Fig. 1b.
